# A New Approach for Estimating Liver IVIM Fast Diffusion Coefficient in Two‐Compartment Model and Fast Diffusion Fraction in Three‐Compartment Model: Theory and In Vivo Validation of Two Centers' Data

**DOI:** 10.1002/nbm.70221

**Published:** 2025-12-29

**Authors:** Fu‐Zhao Ma, Yì Xiáng J. Wáng

**Affiliations:** ^1^ Department of Imaging and Interventional Radiology, Faculty of Medicine The Chinese University of Hong Kong Hong Kong SAR China

**Keywords:** curve fitting, intravoxel incoherent motion, liver, reproducibility, tri‐exponential model

## Abstract

The intravoxel incoherent motion (IVIM) three‐compartment model (3CM) potentially offers better classification for perfusion‐rich pathologies. However, 3CM is often associated with less fitting stability than the two‐compartment model (2CM). For liver intravoxel IVIM data, when the fit‐starting *b*‐value is adjusted from 2 s/mm^2^ to *b*_*i*_ s/mm^2^, *PF*_*i*_, which indicates the perfusion fraction at *b*_*i*_, gradually decreases as the initial fitting *b*‐value *b*_*i*_ increases and eventually approaches zero. We define *Y*_*i*_ = ln [(1 − *PF*_*i*_)/*PF*_*i*_]. With a fitted line between *Y*_*i*_ and *b*_*i*_, the intercept with the *y*‐axis is the predicted value of *Y*_0_ that follows the bi‐exponential model. The *PF*_0_ calculated from it eliminates the signals of other very rapid perfusion components and retains a more stable perfusion fraction measurement. The slope of the fitted line is (*D*_*fast*_–*D*_*slow*_). *D*_*slow*_ can be calculated by the standard IVIM model. Because the *Y*_*i*_ fitting assumes that the signal attenuation is bi‐exponential, and the portion of the signal strength that exceeds the bi‐exponential model at very low *b*‐values is not used, the perfusion fraction detected is the fast decay perfusion component (*F*_*fast*_) without the very fast decay perfusion component (*F*_*vfast*_). In our study on healthy livers, *Dataset‐1* with 3.0 T data had 17 subjects scanned twice with a 10–12 days' interval, and paired scan–rescan had 13 subjects; *Dataset‐2* with 1.5 T data had 20 subjects scanned twice during the same scan session, and paired scan–rescan had 17 subjects. For the 2CM, IVIM parameters *D*_*slow*_, *D*_*fast*_, and *PF*_*i*_ were obtained with a nonlinear least square (NLLSQ) fit. Parameters obtained with segmented fitting were used for *Y*_*i*_ analysis. Results show that conventional fitting and *Yi* fitting derived very comparable IVIM parameters in mean value. *D*_*fast*_ obtained with *Y*_*i*_ fitting showed better scan–rescan stability than conventional segmented fitting. Perfusion fraction parameters showed more favorable scan–rescan stability with *Y*_*i*_ fitting.

## Graphical abstract

When *b*‐value is adjusted from 0 to *b*_*i*_ s/mm^2^, *PF*_*i*_, which indicates the actual perfusion fraction at *b*_*i*_, shifts accordingly. We define *Y*_*i*_ = ln [(1 − *PF*_*i*_)/*PF*_*i*_]. Conventional fitting and *Yi* fitting derived comparable IVIM parameters in mean value. *D*_*fast*_ obtained with *Y*_*i*_ fitting showed better scan–rescan stability than conventional fitting. Perfusion fractions showed favorable scan–rescan stability with *Y*_*i*_ fitting. *F*_*fast*_: second fast compartment related to tri‐exponential model. *F*_*Vfast*_: very fast compartment related to tri‐exponential model. *F*_*tot*_: sum of *F*_*fast*_ and *F*_*Vfast*_.

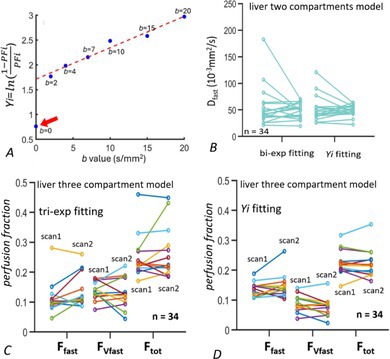

**Abbreviations**
biex: bi‐exponential model
CoV: coefficient of variation
*D*_*fast*_
: the perfusion‐related diffusion coefficient related to bi‐exponential decay model
*D*_*slow*_
: the diffusion coefficient representing the slow (“pure”) molecular diffusion related to bi‐exponential decay model
DW: diffusion weighted
*F*_*fast*_
: fraction of the second fast perfusion‐related diffusion compartment related to tri‐exponential decay model
*F*_*tot*_
: the sum of *F*_*fast*_ and *F*_*vfast*_
*F*_*vfast*_
: fraction of the very fast perfusion‐related diffusion compartment related to the tri‐exponential decay model
IVIM: intravoxel incoherent motion
NSA: number of signal averaging
*PF*
: fraction of the perfusion‐related diffusion compartment related to bi‐exponential decay model
ROIs: regions of interest
SNR: signal‐to‐noise ratio
triex: tri‐exponential model
wSD: within‐subject standard deviation

In diffusion‐weighted (DW) MRI, intravoxel incoherent motion (IVIM) theory was proposed to account for the effect of vessel/capillary perfusion on the aggregate DW MR signal. The fast component of diffusion is related to micro‐perfusion, whereas the slow component is linked to molecular diffusion. The prevalent IVIM modeling is based on Equation (1):

(1) $$S\left(b\right)=S_{0}\cdot \left(\mathrm{PF}\cdot e^{-bD_{\text{fast}}}+\left(1-\mathrm{PF}\right)\cdot e^{-bD_{\text{slow}}}\right)$$

where *S*(*b*) and *S*_0_ denote the signal intensity of images acquired with the *b*‐factor value of *b* and *b* = 0 s/mm^2^, respectively. Three parameters can be computed. *D*_*slow*_ (or *D*) is the diffusion coefficient representing the slow “pure” molecular diffusion (unaffected by perfusion). The perfusion fraction (*PF*, or *f*) represents the fraction of the compartment related to (micro)circulation, which can be understood as the proportional “incoherently flowing fluid” (i.e., blood) volume. *D*_*fast*_ (or D*) is the perfusion‐related diffusion coefficient representing the incoherent microcirculation within the voxel, which holds information for blood perfusion's speed [1, 2]. The signal decay of IVIM diffusion MRI is therefore commonly described with a bi‐exponential (biex) model. Liver is histologically unique in many aspects, which include the presence of several vessel types (arteries/arterioles, portal veins/venules, hepatic veins/venules), sinusoid capillaries, bile ducts, a rich lymphatic system, and a functionally important intermediate area between the sinusoids and hepatocytes (space of Disse). For liver tissue, Cercueil et al. demonstrated that a third very fast diffusion compartment may exist, though the precise origin of this third compartment could not be precisely defined, and the tri‐exponential (triex) model provided the better fit for IVIM signal decay over the *b* = 0 ~ 800 s/mm^2^ range [3, 4]. Wurnig et al. [5] investigated an extensive DW‐imaging protocol including 68 *b*‐values and computed apparent diffusion coefficient “spectra”; they also demonstrated the presence of a third component of diffusion in liver and kidney. Three compartments (very fast compartment, fast compartment, and slow compartment) of the IVIM are modeled according to [3, 6]

(2) $$\begin{array}{c} S\left(b\right)=S_{0}\cdot \left(F_{\text{slow}}\cdot e^{-bD_{\text{slow}}}+F_{\text{fast}}\cdot e^{-bD_{\text{fast}}}+F_{\text{vfast}}\cdot e^{-bD_{\text{vfast}}}\right) \end{array}$$

where *D*_*slow*_ represents the diffusion coefficient, and *D*_*fast*_ and *D*_*vfast*_ represent the fast and very fast perfusion‐related pseudo‐diffusion coefficients. *F*_*slow*_, *F*_*fast*_, and *F*_*vfast*_ are the fractions of each compartment. The combination of *F*_*fast*_ + *F*_*vfast*_ is the *PF* of the biex model, and *F*_*slow*_ is the fraction of the slow diffusion compartment (1 − *PF*). Theoretically, the three‐compartment model (3CM) can improve data fitting precision and may help measure the true multiple‐compartment nature of *in* *vivo* physiology and thus potentially offer better classification for various pathologies [7, 8, 9, 10]. In addition to the liver [3, 6, 10], 3CM has been tested for the brain [7], the kidneys [5, 8], and the placenta during women pregnancy [9]. Efforts on DW data acquisition have been made to improve the fitting results for the 3CM model [11, 12, 13]. However, because of its fitting instability [6], the 3CM has not been popular in application [14, 15].

Even for the two‐compartment model (2CM), fitting stability for the fast compartment remains a concern, particularly for the metric *D*_*fast*_ [6, 16, 17, 18, 19, 20]. Because of the existence of the unstable, very fast component, it has been empirically suggested that *b* = 0 image data can be excluded for liver biex IVIM curve fitting [14, 21, 22, 23, 24]. With fitting starting from a nonzero low *b*‐value, the relationship between DWI signal and *b*‐value better follows a biex decay pattern [22]. With this empirical approach, good results have been reported with IVIM to detect early‐stage liver fibrosis [21, 25, 26]. To expand these reports, this study further investigates from which low *b*‐value fitting starts can ensure better conformity with the biex‐IVIM model. Moreover, a new approach of calculating *D*_*fast*_ coefficient with 2CM and *F*_*fast*_ with the 3CM of liver IVIM is proposed in the current study, which shows higher robustness than the conventional fitting models.

## 1 Materials and Methods

### 1.1 Theory and Fitting Methods

The commonly used IVIM model is described by Equation (1). Signal intensity at a specific *b*‐value *b*_*i*_ equals

(3) $$\begin{array}{c} S\left(b_{i}\right)=S_{0}\cdot \left(\mathrm{PF}\cdot e^{-b_{i}D_{\text{fast}}}+\left(1-\mathrm{PF}\right)\cdot e^{-b_{i}D_{\text{slow}}}\right) \end{array}$$

Assuming the signal decay starts from *b*_*i*_, we can rewrite Equation (1) as

(4) $$\begin{array}{c} S\left(b\right)=S_{0}\cdot \left(\mathrm{PF}\cdot e^{-b_{i}D_{\text{fast}}}\cdot e^{-\left(b-b_{i}\right)D_{\text{fast}}}+\left(1-\mathrm{PF}\right)\cdot e^{-b_{i}D_{\text{slow}}}\cdot e^{-\left(b-b_{i}\right)D_{\text{slow}}}\right) \end{array}$$

To simplify Equation (4), we can do the following normalization:

(5) $$\begin{array}{c} S\left(b\right)=S\left(b_{i}\right)\cdot \left(\begin{array}{c} \frac{\mathrm{PF}\cdot e^{-b_{i}D_{\text{fast}}}}{\mathrm{PF}\cdot e^{-b_{i}D_{\text{fast}}}+\left(1-\mathrm{PF}\right)\cdot e^{-b_{i}D_{\text{slow}}}}\cdot e^{-\left(b-b_{i}\right)D_{\text{fast}}}\\ +\frac{\left(1-\mathrm{PF}\right)\cdot e^{-b_{i}D_{\text{slow}}}}{\mathrm{PF}\cdot e^{-b_{i}D_{\text{fast}}}+\left(1-\mathrm{PF}\right)\cdot e^{-b_{i}D_{\text{slow}}}}\cdot e^{-\left(b-b_{i}\right)D_{\text{slow}}} \end{array}\right) \end{array}$$

The coefficient before the exponential term can be regarded as the new *PF*_*i*_ and (1 − *PF*_*i*_) when we assume that S(b) decays from *b*_*i*_, where

(6) $$\begin{array}{c} PF_{i}=\frac{\mathrm{PF}\cdot e^{-b_{i}D_{\text{fast}}}}{\mathrm{PF}\cdot e^{-b_{i}D_{\text{fast}}}+\left(1-\mathrm{PF}\right)\cdot e^{-b_{i}D_{\text{slow}}}} \end{array}$$

(7) $$\begin{array}{c} 1-PF_{i}=\frac{\left(1-\mathrm{PF}\right)\cdot e^{-b_{i}D_{\text{slow}}}}{\mathrm{PF}\cdot e^{-b_{i}D_{\text{fast}}}+\left(1-\mathrm{PF}\right)\cdot e^{-b_{i}D_{\text{slow}}}} \end{array}$$

*PF*_*i*_ is the actual measured perfusion fraction at *b*‐value = *b*_*i*_ s/mm^2^. We can rewrite Equation (4) in a simple form:

(8) $$\begin{array}{c} S\left(b\right)=S\left(b_{i}\right)\cdot \left(PF_{i}\cdot e^{-\left(b-b_{i}\right)D_{\text{fast}}}+\left(1-PF_{i}\right)\cdot e^{-\left(b-b_{i}\right)D_{\text{slow}}}\right) \end{array}$$

This is the modified IVIM model where the initial fitting *b*‐value varies from 0 to *b*_*i*_ s/mm^2^. It is important to note that Equation (8) still conforms to the biex model, maintaining the same decay rates, *D*_*fast*_ and *D*_*slow*_. Therefore, the fitting methods used for the standard IVIM can also be applied to fitting started from *b*_*i*_, converting the *b*‐value array from *b* to (*b*–*b*_*i*_). Equations (8) and (1) represent different forms of the same equation. When the starting *b*‐value is adjusted from 0 to *b*_*i*_ s/mm^2^, the two exponential decay terms shift accordingly. The parameters *D*_*slow*_ and *D*_*fast*_, which are associated with the velocity of water molecule motion, remain constant. However, *PF*, representing the perfusion fraction, changes to *PF*_*i*_, indicating the actual perfusion fraction at *b*_*i*_. Given that *D*_*fast*_ is much larger than *D*_*slow*_, *PF*_*i*_ will gradually decrease as the initial fitting *b*‐value *b*_*i*_ increases, eventually approaching zero, signifying the decay of the perfusion component. Taking the logarithm of Equation (6), we can rewrite that as

(9) $$\begin{array}{c} \ln\left(\frac{1-PF_{i}}{PF_{i}}\right)=b_{i}\left(D_{\text{fast}}-D_{\text{slow}}\right)+\ln\left(\frac{1-\mathrm{PF}}{\mathrm{PF}}\right) \end{array}$$

Parameters *D*_*slow*_, *D*_*fast*_, and *PF* are constants reflecting the property of liver tissue. *PF*_*i*_ is a fitted parameter that varies with the initial fitting *b*‐value *b*_*i*_. When *b*_*i*_ = 0, the fitted *PF*_*i*_ is the measurement of *PF*. We define

(10) $$\begin{array}{c} Y_{i}=\ln\left(\frac{1-PF_{i}}{PF_{i}}\right) \end{array}$$

By establishing an initial value for *b*_*i*_, the corresponding *PF*_*i*_ and *Y*_*i*_ at each *b*‐value can be determined by applying the standard IVIM fitting method. When the initial fitting *b*‐value is increased in steps, a series of *b*_*i*_ and their corresponding *PF*_*i*_ *, Y*_*i*_ values can be obtained. As illustrated in Equation (9), a linear relationship between *Y*_*i*_ and *b*_*i*_ will be observed if *S* (*b*_*i*_), the signal intensity at *b*_*i*_, follows the predicted biex decay model. Once this relationship has been confirmed, a fitted line between *Y*_*i*_ and *b*_*i*_ can be used to reflect the IVIM parameters. The intercept of the fitted line with the *Y*‐axis is the predicted value of *Y*_0_ that follows the bi‐exponential model. The *PF*_0_ calculated from this intercept eliminates the signals from other rapidly decaying perfusion components and retains the relatively stable perfusion fraction measurements. For the 3CM, the calculated *PF*_0_ here represents the perfusion fraction after excluding the very fast component, denoted as *F*_*fast*_. However, if we initiate the fitting from *b*‐value = 0 and consider all perfusion components with different diffusion rates as a whole, fitting with Equation (1) yields a value for *PF*_0_ that encompasses the entire perfusion fraction—i.e., *F*_*tot*_ in the 3CM framework (though the resulting *D*_*fast*_ value with this fitting is inevitably inaccurate, as it effectively represents a composite of multiple diffusion coefficients). By taking the difference between *F*_*tot*_ and *F*_*fast*_, we can derive *F*_*vfast*_. This approach allows us to estimate three parameters of the 3CM without performing a tri‐exponential fit.

According to Equation (9), the slope of the fitted line is (*D*_*fast*_–*D*_*slow*_). *D*_*slow*_ can be directly obtained by a mono‐exponential fit to the signal for a *b*‐value of > 60 s/mm^2^ (same method used for IVIM Segmented fitting) [14, 24] and is independent of our new method. Based on this, we can derive a more precise estimate of *D*_*fast*_, which is comprehensively weighted by multiple *b*‐values rather than being restricted by the initial fitting *b*‐value.

### 1.2 DW MRI Image Data Acquisition

**FIGURE 1:**
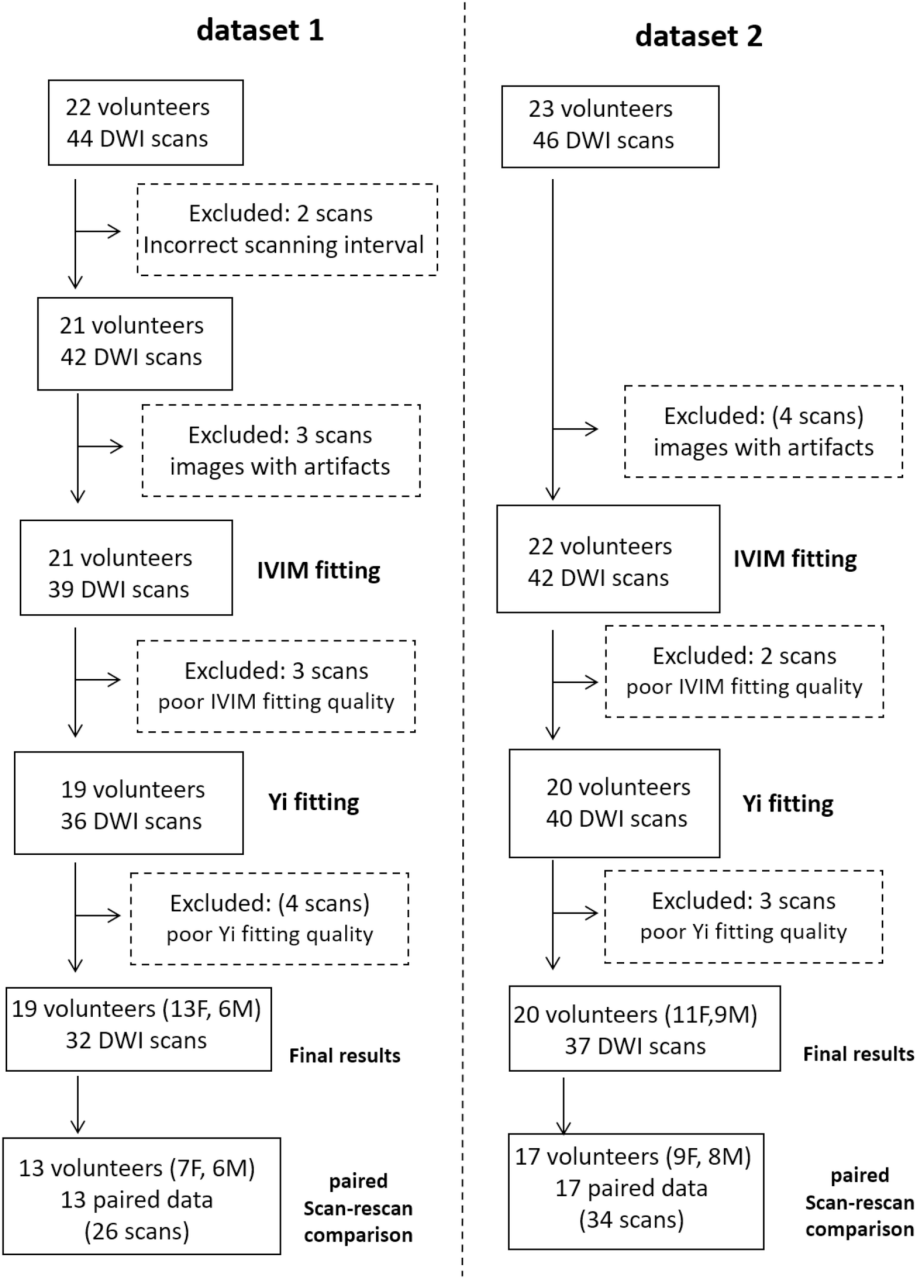
Liver DWI data inclusion process. *Dataset‐1* was from the work published by Li et al. [23], and *Dataset‐2* was from the work published by Huang et al. [24]. The liver DWI of Li et al. had 22 healthy volunteers scanned twice for scan–rescan reproducibility assessment, and we aimed to include those with 10–12 days' interval between the two scans. For *Dataset‐2*, the MRI data of the initial 23 healthy volunteers scanned twice during the same scan session were chosen for the current study (i.e., 23 pairs of scanned were chosen chronologically). Images with notable motion and artifacts were first discarded [27]. A small portion of images with poor IVIM data fitting (as described in [27]) or with poor *Y*_*i*_ fitting were further excluded (examples provided in Figure S1). For *Dataset‐1*, final results had 13 females (age range: 23–58 years, mean: 27.92 years) and six males (age range: 24–31 years, mean: 25.83 years). Paired scan–rescan comparison had seven females (age range: 23–26 years, mean: 25.14 years) and six males (age range: 24–31 years, mean: 25.83 years). For *Dataset‐2*, final results had 11 females (age range: 20–62 years, mean: 42.09 years) and nine males (age range: 25–60 years, mean: 41.56 years). Paired scan–rescan comparison had nine females (age range: 20–62 years, mean: 43.11 years) and eight males (age range: 25–55 years, mean: 39.25 years).

Two previously acquired healthy volunteer liver IVIM data were reused in the current study; images were acquired at 3.0 T [23] and 1.5 [24], respectively. The healthy volunteer upper abdomen MRI data acquisition as observational studies was approved by the institutional ethical committee, and informed consent was obtained for all subjects. The study participants were all known to be healthy at the MRI exam and at the 6‐month follow‐up after the exam, without liver, spleen, and other abdominal organ disease history, and not on any regular medication. Specific MR imaging data acquisition parameters are shown in Table 1. To assess scan–rescan agreeability, for *Dataset‐1*, each subject attended two scan sessions with an interval of 10–20 days; for *Dataset‐2*, each subject was scanned twice during the same session. The data enrolment process is shown in Figure 1.

**TABLE 1:** MR imaging parameters for *Dataset‐1* and *Dataset‐2*.

	Dataset 1	Dataset 2
Vendor	Phillips	Phillips
Field strength (*T*)	3.0	1.5
Sequence name	Single‐shot FSE EPI	Single‐shot FSE EPI
Repetition time (ms)	1600	1600
Echo time (ms)	59	57.8
Number of slices	20	6
Slice thickness (mm)	7	7
Slice intergap (mm)	1.5	1
Field of view (mm^2^)	350 × 372	375 × 302
Matrix	116 × 124	124 × 97
Parallel acceleration factor	SENSE = 4	SENSE = 3
Fat saturation	SPIR	SPIR
Echo train length	39	35
Scan duration (min)	5.4	9.9
Time interval between two scans	10 ~ 20 days	Consecutive
*b*‐values (s/mm^2^)^a^	0, 2, 4, 7, 10, 15, 20, 30, 46, 60, 72, 100, 150, 200, 400, 600. (16 *b*‐values)	0, 2, 4, 7, 10, 15, 20, 30, 46, 60, 72, 100, 150, 200, 400, 600. (16 *b*‐values)
Number of excitations at each *b*‐value	3 (*b* = 0, 2, 60), 2 (*b* = 400, 600), 1 (*b* = 4, 7, 10, 15, 20, 30, 46, 72, 100, 150, 200)	2 at all *b*‐values

^a^ *b*‐values are nominal values.

### 1.3 DW MRI Image Data Analysis

**FIGURE 2:**
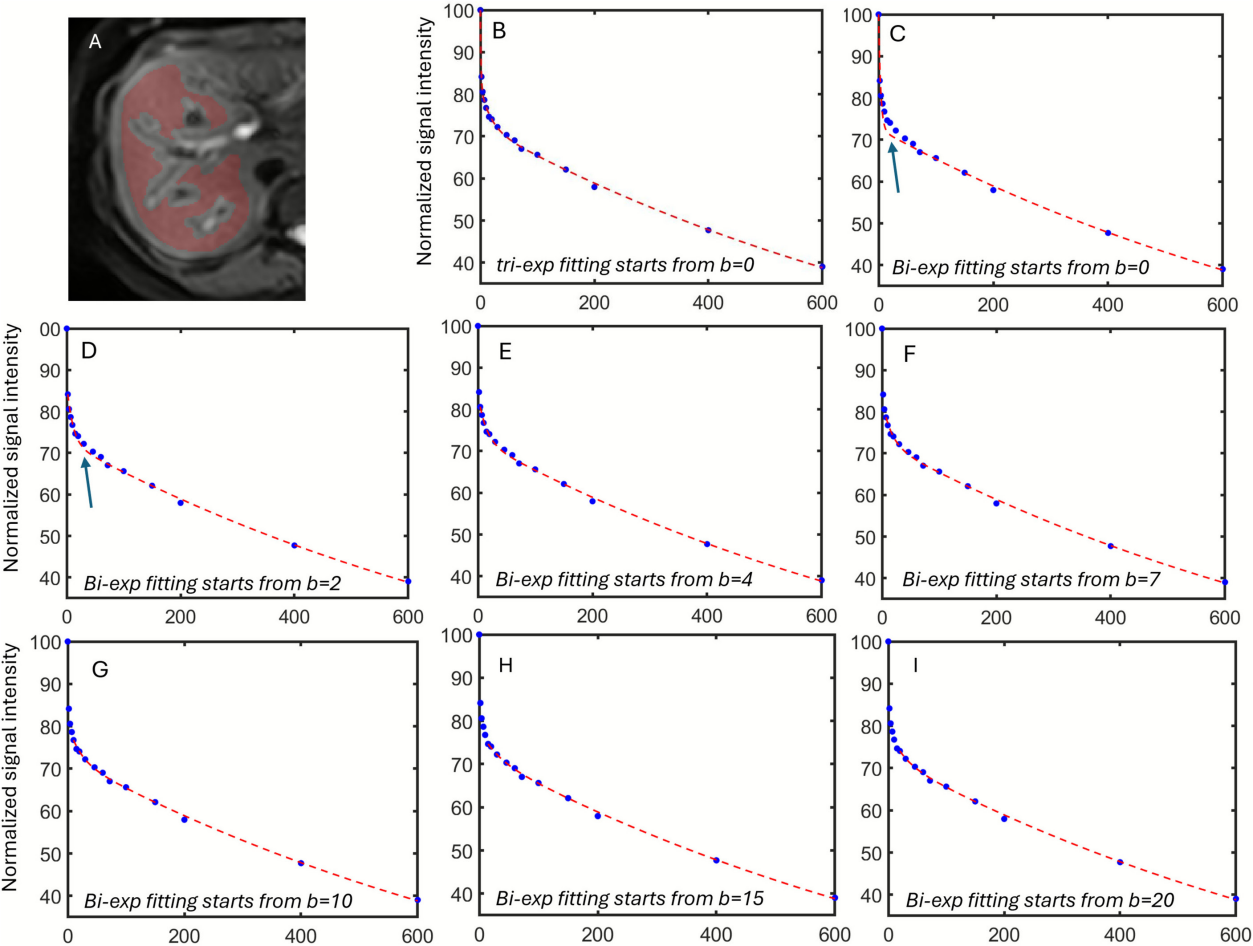
Example of IVIM fitting curves. (A) ROI was shown on the DW image to cover a large portion of the right liver parenchyma while avoiding large vessels. (B) Data points with all the 16 *b*‐values were utilized for triex fitting (from *b* = 0 to *b* = 600 s/mm^2^), showing good fitting quality. (C–I) Biex fitting curves of one DWI scan in this study with varied fit‐starting *b*‐value *b*_*i*_ (from *b* = 0 to *b* = 20 s/mm^2^). In (B), when biex fitting was conducted starting from *b* = 0 s/mm^2^, a marked fitting imperfection is noted (arrow). In (C), when biex fitting was conducted starting from *b* = 2 s/mm^2^, a mild fitting imperfection is still noted (arrow), indicating a small portion of a very fast component. In (D–I), when biex fitting was conducted starting from *b* = 4, 7, 10, 15, or 20 s/mm^2^, the fitted curves resemble a biex fitting decay curve with good fitting.

Segmentation of the region of interest (ROI), curve fitting, and parameter estimation were conducted using in‐house codes written in MATLAB (MathWorks, Natick, MA, USA). ROIs were placed on a *b* = 0 s/mm^2^ image to cover a large portion of the right liver parenchyma while avoiding large vessels and then copied to the images of other *b*‐values of this slice. The coverage of the ROI over the slices other than the *b* = 0 s/mm^2^ image was checked, and when respiration‐associated liver positioning shift or other artifacts were noted for a slice, then the ROI on that slice was manually edited so that the ROI properly covered liver parenchyma, as shown in Figure 2A.

The *Y*_*i*_ analysis was based on the standard IVIM fitting model. First, one specific *b*‐value was chosen as the fit‐starting *b*‐value, and that was in order of 0, 2, 4, 7, 10, 15, and 20 s/mm^2^ in this study. The *b*‐values equal to or larger than these values were utilized for standard IVIM segmented fitting with their corresponding DW image signal intensity. The examples of fitting curve started from different *b*‐values are shown in Figure 2B–I. In each fit, *D*_*slow*_, *D*_*fast*_, and *PF*_*i*_ were free parameters; *S* (*b*_***i***_) was fixed and used for normalization.

For conventional IVIM fitting with the nonlinear least squares fitting (NLLSQ) method, both full fitting and segmented fitting were applied. Full fitting was used to show the trend of the *PF*_*i*_ change following the starting fitting *b*‐value (*b*_*i*_) increases. Three IVIM parameters *D*_*slow*_, *D*_*fast*_, and *PF*_*i*_ were obtained in one NLLSQ fit after normalization using *S* (*b*_*i*_). Segmented fitting was used for *Y*_*i*_ analysis, and the threshold *b*‐value to separate the fast component and slow component was 60 s/mm^2^ as described previously [23, 24]. When the *b*‐value is larger than that threshold, we assume that the perfusion‐related components have decayed to be immeasurable, and the signal intensity attenuation follows a mono‐exponential decay with *b*‐values. Therefore, after logarithmic transformation, *D*_*slow*_ and *PF*_*i*_ can be calculated with a linear fit. The obtained results were used in a constrained NLLSQ fit to determine the value of *D*_*fast*_ [6]. Each IVIM segmented fitting at one initial fitting *b*‐value *b*_*i*_ led to a corresponding set of *PF*_*i*_ and *D*_*fast*_ values. The resulting *PFᵢ* values, obtained across a range of increasing *bᵢ* values, were then used to calculate their corresponding *Yᵢ* values using Equation (10). Finally, a linear regression was performed on the paired data points (*bᵢ*, *Yᵢ*). The array of *b*_*i*_ and *Y*_*i*_ was (*b*_0_, *Y*_0_), (*b*_2_, *Y*_2_), (*b*_4_, *Y*_4_), (*b*_7_, *Y*_7_), (*b*_10_, *Y*_10_), (*b*_15_, *Y*_15_), and (*b*_20_, *Y*_20_).

The result of *Y*_*i*_ linear fitting is employed for parameter estimation as shown in Equation (9): The gradient of the fitted line represents the difference between *D*_*fast*_ and *D*_*slow*_, as previously explained. The *Y*‐intercept of the line provides the predicted value of *Y*_0_ that follows the bi‐exponential model. The *PF*_0_ calculated by this approach eliminates the signals of other rapidly decaying perfusion components and retains the relatively stable perfusion fraction measurements. Our new method assumes that signal attenuation is bi‐exponential. The portion of signal strength exceeding the bi‐exponential at very low *b*‐values was not used for parameter calculations. Therefore, the perfusion fraction we can detect is about the fast decay perfusion component (*F*_*fast*_) without the very fast decay perfusion component (*F*_*vfast*_).

Triex‐IVIM model was used to test scan–rescan stability of the measurements of *PF* calculated from the intercept. Full fitting method was used, which means that all the tri‐exponential parameters (*D*_*slow*_, *D*_*fast*_, *D*_*Vfast*_, *F*_*slow*_, *F*_*fast*_, and *F*_*Vfast*_, 6 free parameters) were obtained through one NLLSQ fit while *S*(0) was fixed and used for normalization.

Statistical analysis was implemented in GraphPad Prism Software (GraphPad Software Inc., San Diego, CA, USA). To assess the goodness‐of‐fit for the linear fit of *Y*_*i*_, the coefficient of determination (*R* ^2^) and residuals were calculated. The residual at each fitted *b*‐value was defined as the actual value minus the predicted value. The smaller the residual, the closer the measured value is to the predicted value. In this study, the smaller the residuals of *Y*_*i*_ at a certain *b*‐value, the stronger the linear relationship between it and *b*_*i*_ (as demonstrated in Equation 9). A smaller residual implies that the measured *Y*_*i*_ (or *PF*_*i*_) at that point is more closely aligned with the prediction of the biex‐IVIM model. Scan–rescan stabilities for parameter estimation were assessed by the within‐subject standard deviation (wSD), Bland–Altman (B‐A) mean difference and B‐A 95% limits of agreement, and intraclass correlation coefficient (ICC).

## 2 Results

### 2.1 *PF*_*i*_ Decreases Following the Increase of Fit‐Starting *b*‐Value, and the CoV Results Show *b*_*i*_ ≤ 10 s/mm^2^ Is Favored Over Larger *b*_*i*_

**FIGURE 3:**
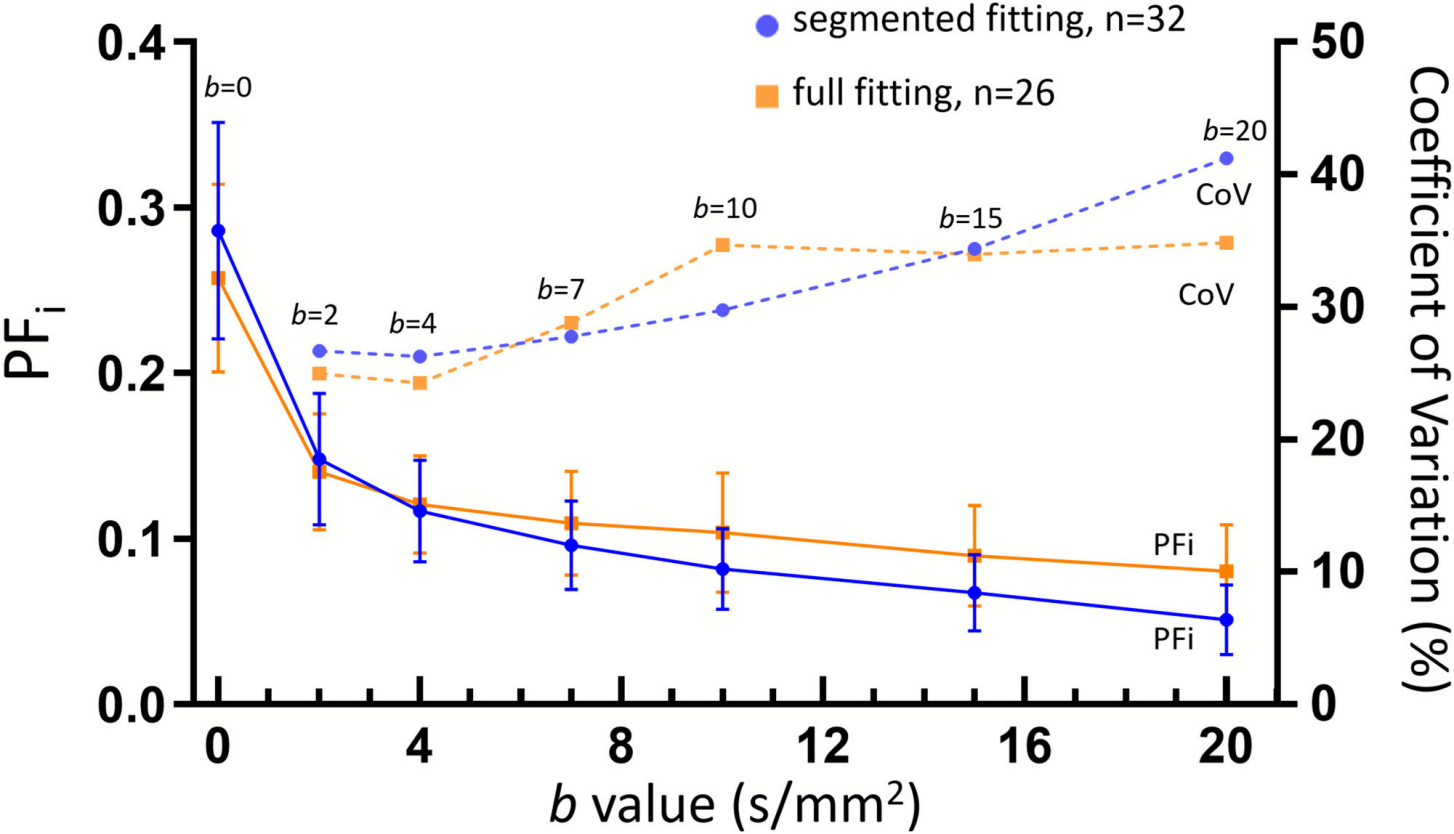
Variation (mean ± standard deviation) of *PF*_*i*_ and its CoV (coefficient of variation) after changing the fit‐starting *b*‐value *b*_*i*_. The blue data points denote the segmented fitting results, whereas the orange data points denote the full fitting results. The solid lines represent the variation of *PF*_*i*_, and the broken lines represent the variation of CoV of the corresponding *PF*_*i*_. The results are based on *Dataset‐1* (*n* = 32 scans).

The change in conventional biex‐IVIM *PF*_*i*_ following the increase of fit‐starting *b*‐value is shown in Figure 3. As predicted by Equation (6), the measured values of *PF*_*i*_ gradually decreased as the fit‐starting *b*‐value increased. For segmented fitting, *PF*_*i*_ decreased from 0.280 ± 0.126 at *b_i_* = 0 to 0.145 ± 0.076 at *b_i_* = 2 s/mm^2^, reaching 0.051 ± 0.041 at *b*_*i*_ = 20 s/mm^2^. Notably, when the starting *b*‐value increased from 0 to 2 s/mm^2^, *PF*_*i*_ substantially decreased. In this study, the CoV at *bᵢ* = 2 s/mm^2^ was 26.7% for segmented fitting and 25.0% for full fitting. In contrast, at *bᵢ* = 20 s/mm^2^, the CoV increased to 41.2% for segmented fitting and 34.8% for full fitting. This suggests a smaller starting *b*‐value is associated with better measurement stability. Given the general consistency observed between segmented fitting and full fitting and considering the known relative robustness of segmented fitting as compared to full fitting [4, 8], for subsequent analyses, we only used the results based on segmented fitting for biex‐IVIM.

### 2.2 Linear Relationship Between *b*_*i*_ and *Y*_*i*_ in the Range of *b*_*i*_ Between 2 and 20 s/mm^2^

**FIGURE 4:**
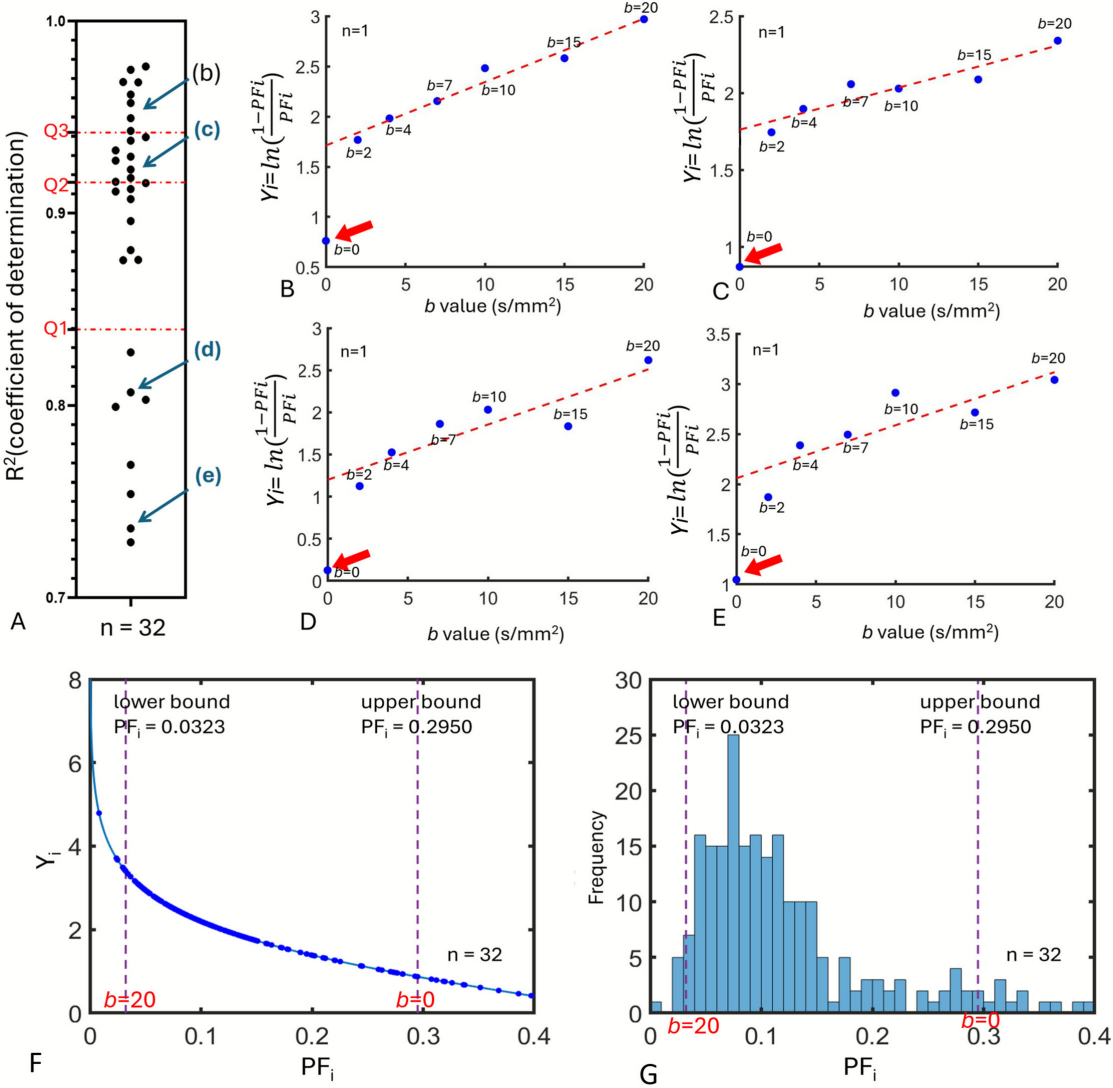
Linear regression results of *Y*_*i*_ when fitted with *b*_*i*_ = 2, 4, 7, 10, 15, and 20 s/mm^2^. *R* ^*2*^ of the *Y*_*i*_ fitting for *Dataset‐1* are shown in (A), where each point corresponds to one DWI scan's result. The three dashed lines correspond to the three quartiles of the distribution: Q1 (first quartile), Q2 (median), and Q3 (third quartile). Dots denoted with (b), (c), (d), and (e) in (A) are results with different *R* ^*2*^ qualities [fit results for the four scans shown in (B–D)]. For the four scans illustrated in (B–E), (B) was the best in the quality of fitting, followed by (C), then by (D), and then by (E). Regardless of the fitting quality as shown in (A), six *Y*_*i*_ values corresponding to *b*_*i*_ = 2, 4, 7, 10, 15, and 20 s/mm^2^ can be linearly fitted, whereas the *Y*_*i*_ value corresponding to *b*_*i*_ = 0 s/mm^2^ does not correlate with the linear fitting results of other *b*‐values (shown by the thick red arrow). The results in (A–E) show the rationale of not including *b* = 0 for *Y*_*i*_ fitting. (F) A numerical simulation of the function of *Y*_*i*_ with independent variable *PF*_*i*_, and with the value of *Y*_*i*_ constrained below 8. The relationship between *Y*_*i*_ and *PF*_*i*_ is not linear, especially when *PF*_*i*_ is very small. (G) The histogram of the actual *PF*_*i*_ values in this study for analysis for *Y*_*i*_. Bar width = 0.01. *Y*‐axis frequency means the number of *PF*_*i*_ located in this bar. Most of the *PF*_*i*_ used for analysis are located at the region where *Y*_*i*_ and *PF*_*i*_ have a relatively linear relationship.

Figure 4A–G for *Dataset‐1* shows the relationship between *b*‐value ranging from 0 to 20 s/mm^2^ and *Y*_*i*_. After excluding the datapoint at *b* = 0, there was a good linear relationship between *b*_*i*_ and *Y*_*i*_. Moreover, at *b* = 0, *Y*_0_ consistently lay substantially below the fitted line, i.e., the measured *PF*_0_ was substantially higher than could be predicted from the IVIM bi‐exponential model. Analysis for *Dataset‐2* showed comparable results (shown in Figure S2).

### 2.3 Including *b* = 0 Incurs *Y*_*i*_ Fitting Instability and PF_2_ Is Associated With a “Small” Extent of Very Fast Component

**FIGURE 5:**
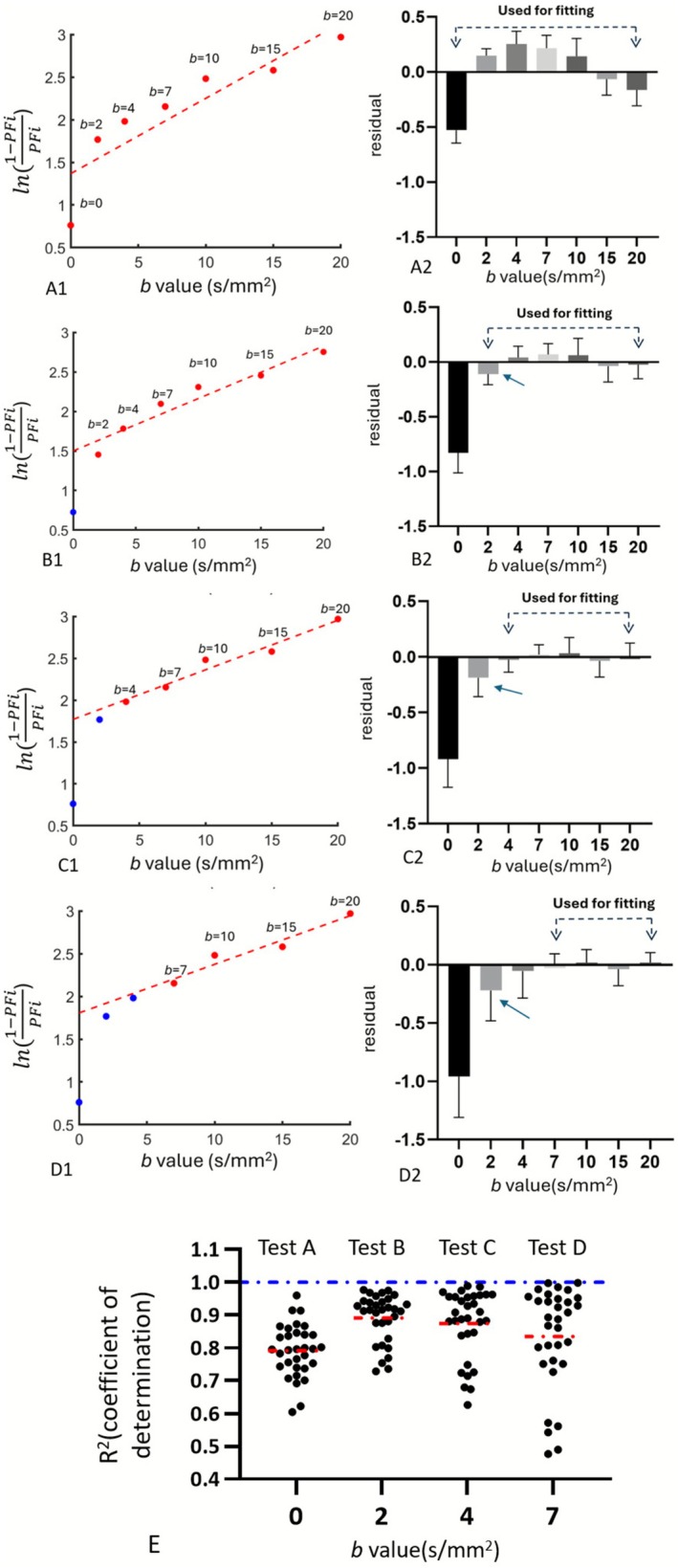
Residuals when varying the number of *b*‐values included in the linear fit between *b*_*i*_ and *Y*_*i*_. (A1), (B1), (C1), and (D1) show the linear fit of *Y*_*i*_ values based on *b* = 0 ~ 20 s/mm^2^, *b* = 2 ~ 20 s/mm^2^, *b* = 4 ~ 20 s/mm^2^, and *b* = 7 ~ 20 s/mm^2^, respectively (blue dots not included for fitting). (A1–D1) All results of a single scan. (A2) Residual corresponding to each *b*‐value (*n* = 7 in total) was calculated scan‐by‐scan according to the linearly fitted line (*n* = 32 scans). For (B2), (C2), and (D2), 6, 5, or 4 *b*‐values were used for the linear fitting, and residuals to such a fitted line were then calculated scan‐by‐scan (*n* = 32 scans). In (A2–D2), the length of the box represents the mean of residuals of all 32 linear fittings, and the bar denotes the 95% CI. In (B2), (C2), and (D2), data points with *b*‐value lower than *b*_*i*_ are not involved in the fit, but a corresponding residual is still calculated by extrapolating the fitted line. Residuals at *b* = 0 are substantially larger than residuals at other *b*‐values. Note that the residual at *b* = 2 s/mm^2^ is larger than other higher *b*‐values illustrated by the blue arrow (B2, C2, D2), suggesting that the signal intensity at *b* = 2 s/mm^2^ exceeds the value predicted by the bi‐exponential model. (E) Varying *R* ^*2*^ (coefficient of determination) of *Y*_*i*_ obtained by fitting from different *b*_*i*_. The *b*‐values used for each group are Test A: 0, 2, 4, 7, 10, 15, and 20 s/mm^2^; Test B: 2, 4, 7, 10, 15, and 20 s/mm^2^; Test C: 4, 7, 10, 15, and 20 s/mm^2^; Test D: 7, 10, 15, and 20 s/mm^2^. Each data point in (E) corresponds to a DWI scan and a linear fit. The blue dashed line: maximum *R* ^*2*^ = 1, the red dashed lines: mean *R* ^2^ values for each group. *R* ^*2*^ values: 0.791 ± 0.028 for Test A, 0.891 ± 0.025 for Test B, 0.871 ± 0.036 for Test C, and 0.835 ± 0.054 for Test D. Thus, Test B exhibits the best linear fit quality, followed by Test C, suggesting that fitting models starting from *b* = 2 s/mm^2^ or *b* = 4 s/mm^2^ align better with the biex‐IVIM model.

According to Equation (10) and as shown in Figure 4, the application of *b*_*i*_ at 0 or 2 s/mm^2^ yielded lower *Y*_*i*_ values, implying an overestimation of *PF*_*i*_ compared with the purely biex decay pattern of the signal. At *b*_*i*_ = 0, *Y*_0_ consistently lay substantially below the fitted line, i.e., the measured *PF*_0_ was substantially higher than that can be predicted from the actual biex model. When *b* = 0 was included for linear fitting between *b*_*i*_ and *Y*_*i*_, the fitting resulted in the largest overall residuals and the smallest *R* ^2^ (Figure 5A). Thus, including *b* = 0 in the results disrupted the linear relationship between *b*_*i*_ and *Y*_*i*_. Figure 5B,C,D further demonstrates that the same phenomenon at a smaller scale was also evident at *b* = 2 s/mm^2^. The residuals at *b* = 2 s/mm^2^ remained consistently negative and significantly larger than at other points. This suggests that *PF*_2_ was also higher than the PF predicted by the biex model for *b* = 2 s/mm^2^, implying that it is also associated with a very fast component (also see Figure S3). Figure 5A2 shows that fitting residuals are relatively larger when fitting is started at *b* = 0 s/mm^2^, whereas fitting that started from *b* = 2 s/mm^2^ shows the best (i.e., the largest) *R* ^2^ (also shown with Figure 5E). Thus, fitting models starting from *b* = 2 s/mm^2^ align best with the biex IVIM model. Analysis for *Dataset‐2* yielded comparable results (shown in Figure S3).

### 2.4 With 2CM, Better Scan–Rescan Agreement for *D*_*fast*_ Estimated From *Y*_*i*_ (From *Y*_2_ to *Y*_20_) Is Obtained Than *D*_*fast*_ Estimated by Conventional Biex Fitting

**FIGURE 6:**
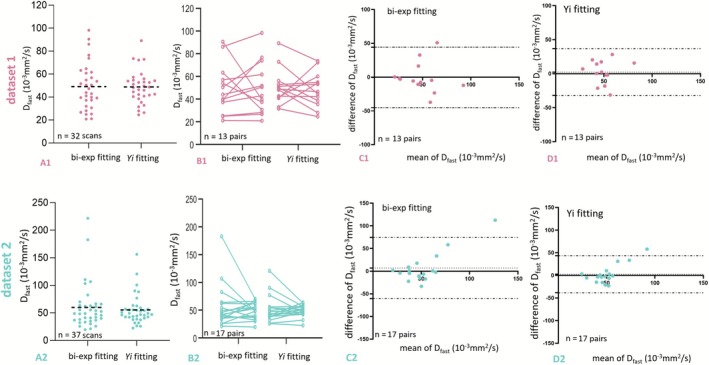
Measurements and scan–rescan agreement (Bland–Altman plot for C1, D1, C2, and D2) for bi‐exponential fitting parameters *D*_*fast*_, two dataset results. Bar in (A1) and (A2) denotes the mean values. Data points utilized for *Y*_*i*_ fitting are *b*_*i*_ = 2, 4, 7, 10, 15, and 20 s/mm^2^.

A better scan–rescan agreement for *D*_*fast*_ estimated from *Y*_*i*_ (from *Y*_2_ to *Y*_20_) than *D*_*fast*_ estimated by conventional biex fitting is shown in Figure 6 and Table 2. Table 2 shows that conventional fitting and *Y*_*i*_ fitting derived comparable *D*_*fast*_ mean values (*Dataset‐1*, 60.0 for conventional fitting, 55.5 for *Y*_*i*_ fitting; *Dataset‐2*, 49.1 for conventional fitting, 48.8 for *Y*_*i*_ fitting, unit in ×10^−3^ mm^2^/s). For both *Dataset‐1* and *Dataset‐2*, *Y*_*i*_ fitting offered smaller CoV, smaller wSD, smaller B‐A difference, and smaller B‐A 95% limit, suggesting substantial improvement in stability with *Y*_*i*_ fitting compared with conventional bi‐exponential fitting (as shown in Figure 6).

**TABLE 2:** Measurements and scan–rescan agreement for the bi‐exponential fitting parameter *D*_*fast*_.

	Dataset 1			Dataset 2		
	Subject no.	Seg fitting	*Y*_*i*_ fitting	Subject no.	Seg fitting	*Y*_*i*_ fitting
Mean	32 scans	49.1	48.8	37 scans	60.0	55.5
CoV	32 scans	0.412	0.295^a^	37 scans	0.685	0.480^a^
wSD	13 pairs	15.516	12.070^a^	17 pairs	24.174	14.493^a^
B‐A difference	13 pairs	−0.476^a^	2.346	17 pairs	6.985	2.499^a^
B‐A 95% limit	13 pairs	−45.23 to 44.28	−32.15 to 36.84^a^	17 pairs	−60.63 to 74.60	−38.60 to 43.60^a^

^a^ The corresponding method has a better performance in stability measurements. The unit of mean, wSD, B‐A difference, and B‐A 95% limit is 10^−3^ mm^2^/s.

### 2.5 With 3CM, Better Scan–Rescan Agreement for Perfusion Fractions Estimated From *Y*_*i*_ Fitting (From *Y*_2_ to *Y*_20_) Is Obtained Than Perfusion Fractions Estimated by Conventional Triex Decay Fitting

**FIGURE 7:**
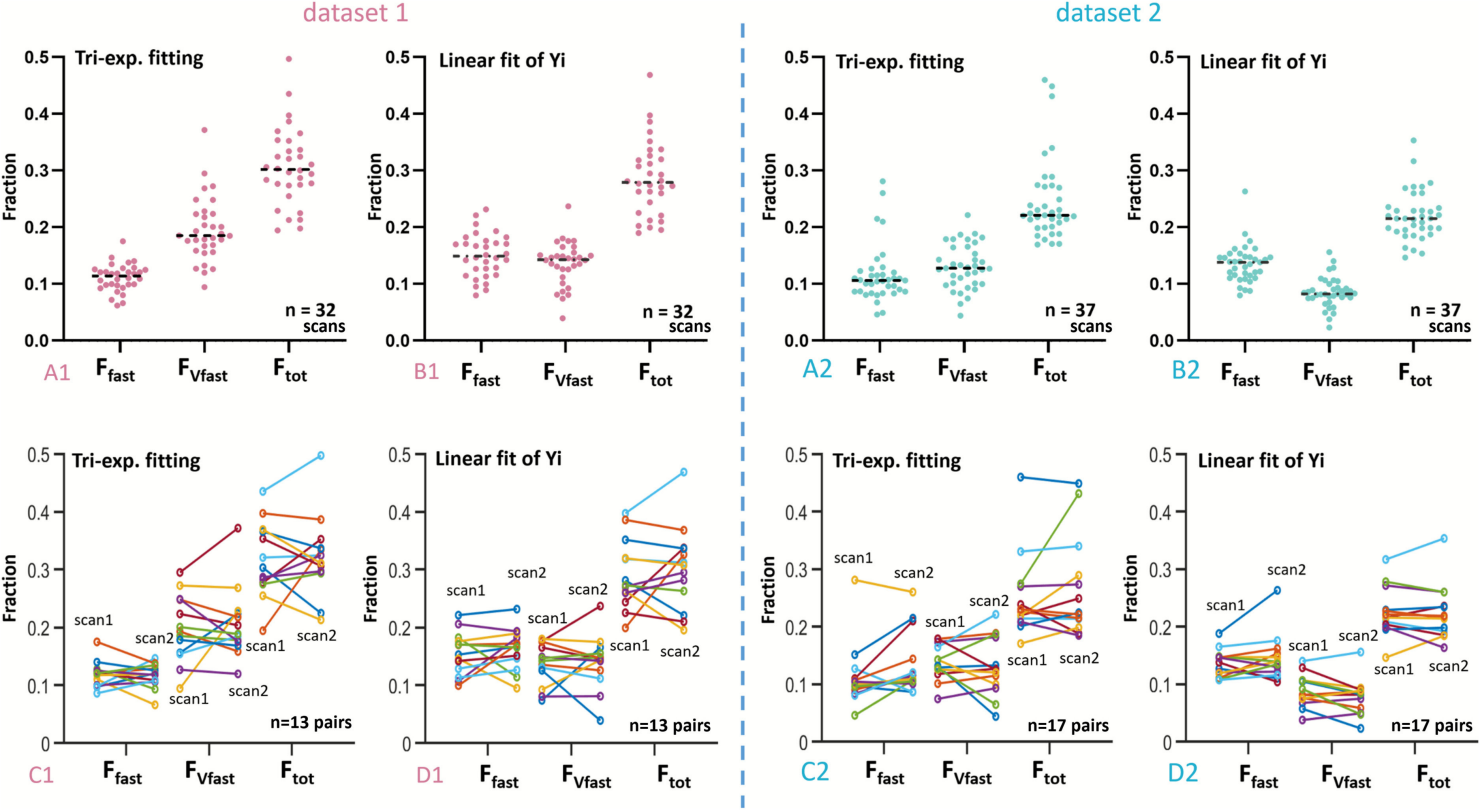
Measurements and scan–rescan agreement for three tri‐exponential fitting parameters *F*_*tot*_, *F*_*Vfast*_, *F*_*fast*_, and two dataset results. Bars in (A1), (B1), (A2), and (B2) denote the mean values. Data points utilized for *Y*_*i*_ fitting are *b*_*i*_ = 2, 4, 7, 10, 15, and 20 s/mm^2^.

When a triex‐IVIM model was considered, the three fraction compartment proportions estimated by *Y*_*i*_ fitting (from *Y*_2_ to *Y*_20_) and conventional fitting are shown in Figure 7. Graphical results suggest that, for *Dataset‐1*, conventional fitting and *Y*_*i*_ fitting derived comparable results in scan–rescan stability, whereas for *Dataset‐2*, measures derived from the conventional fitting were more “scattered” than measures derived from the *Y*_*i*_ fitting. As quantified in Table 3, scan–rescan agreement was comparable with *Y*_*i*_ fitting and conventional fitting for *Dataset‐1*, and scan–rescan agreement was better with *Y*_*i*_ fitting than conventional fitting for *Dataset‐2*. It is noteworthy that scan–rescan stability, as shown in Table 3, with a mean ICC of around 0.7, is remarkably good for 3CM liver IVIM parameters [6].

**TABLE 3:** Measurements and scan–rescan agreement for three tri‐exponential fitting parameters *F*_*tot*_, *F*_*vfast*_, and *F*_*fast*_.

		Dataset 1				Dataset 2			
		Subject no.	*F*_*tot*_	*F*_*vfast*_	*F*_*fast*_	Subject no.	*F*_*tot*_	*F*_*vfast*_	*F*_*fast*_
Triex. fitting	Mean	32 scans	0.306	0.195	0.112	37 scans	0.246	0.130	0.116
	CoV	32 scans	0.223^a^	0.289	0.216^a^	37 scans	0.296	0.303	0.436
	wSD	13 pairs	0.039^a^	0.030^a^	0.027	17 pairs	0.035	0.031	0.028
	ICC	13 pairs	0.646^a^	0.440	0.463^a^	17 pairs	0.788	0.419	0.712^a^
*Y*_*i*_ fitting	Mean	32 scans	0.286	0.136	0.150	37 scans	0.220	0.089	0.131
	CoV	32 scans	0.228	0.283^a^	0.253	37 scans	0.197^a^	0.297^a^	0.230^a^
	wSD	13 pairs	0.042	0.038	0.018^a^	17 pairs	0.016^a^	0.018^a^	0.018^a^
	ICC	13 pairs	0.607	0.591^a^	0.263	17 pairs	0.870^a^	0.574^a^	0.661

^a^ The corresponding method has a better performance in stability measurements.

## 3 Discussion

This study confirms that *PF*_0_ is associated with a substantial contribution of a very fast perfusion component, and the measured value of *PF*_*i*_ gradually decreases as the fit‐starting *b*‐value increases. Fitting quality *R* ^2^ also favors *PF*_*i*_ starting from *b* = 2 s/mm^2^ or *b* = 4 s/mm^2^ (Figures 2 and 4), though *PF*_2_ with *b* = 2 s/mm^2^ still contains a smaller portion of a very fast perfusion component contribution. For the physiological evaluation of normal tissue perfusion, it may be meaningful to clearly separate the very fast component and the fast component. However, for the separation of normal tissue and pathological tissues, or for the grading of pathological severity, a clear separation of the very fast component and the fast component may not be necessary; the mixed components *PF*_2_ may be even preferred. In the study of Li et al., where the IVIM *b*‐value distribution was the same as the current study [21], it was empirically shown that biex‐IVIM fitting starting from *b* = 2 s/mm^2^ offers the best separation of healthy volunteers and liver fibrosis patients. With many clinical scanners, the lowest nonzero *b*‐value is 10 s/mm^2^. This study also demonstrates that, when *bᵢ* is larger than 10 s/mm^2^, the accuracy of *PF*_*i*_ decreases progressively, as demonstrated with the increased CoV for *PF*_*i*_ (Figure 3). However, biex‐IVIM fitting starting from the lowest *b*‐value of 10 s/mm^2^ still follows a typical exponential pattern for the liver (Figures 2 and 3). As shown in Figure 4, as long as *b* = 0 s/mm^2^ was excluded for linear fitting, further excluding *b* = 2 and *b* = 4 s/mm^2^ did not lead to major differences in residuals as compared with excluding *b* = 0 s/mm^2^ only. Regardless of the fitting quality, after excluding *b* = 0 s/mm^2^, there was a good linear relationship between *b*_*i*_ and *Y*_*i*_. The results from this study support the empirical application of biex‐IVIM modeling starting from *b* = 10 (or even *b* = 15) s/mm^2^ for the liver, as shown in the studies reported by Wang et al. [25] and Huang et al. [26]. The studies of Wang et al. [25] and Huang et al. [26] used an IVIM analysis with a fit‐starting *b*‐value of 10 and 15 s/mm^2^, in both studies, a total separation of healthy livers and fibrotic livers was achieved.

This study also suggests that fit‐starting *b*‐value > 20 s/mm^2^ is not favored. At higher *b*_*i*_, the measured value of the corresponding *PF*_*i*_ was very small, and the accuracy was reduced because the value was too close to 0. According to Equation (9), this will lead to very large fluctuations in the value of the calculated *Y*_*i*_. According to Figure 4F, there is a good linear relationship between *Y*_*i*_ and *PF*_*i*_ only in the interval where *PF*_*i*_ is greater than 0.05 and less than 0.4 (with Equation 9, the relationship between *Y*_*i*_ and *PF*_*i*_ is also not linear at too high *PF*_*i*_, but this value is much higher than the maximum *PF*_*i*_ that can be practically measured, and therefore not shown in Figure 4F). If the *PF*_*i*_ is too small, its small measurement error will cause a large variation in *Y*_*i*_, so the measurement of these points has a more significant effect on the result of the linear fit. When *b*_*i*_ = 25 s/mm^2^, the mean value of the *PF*_*i*_ measurements would be below 0.03, violating the above‐mentioned principles. The results in Figures 4 and 5 confirm that there was indeed a linear relationship between *b*_*i*_ and *Y*_*i*_ after removing the very fast perfusion component. This also indicates that the conclusion in Equation (9) is valid, so we used this as a basis for the measurement of IVIM‐related parameters.

In conventional biex‐IVIM fitting, good scan–rescan reproducibility can be achieved when careful data acquisition and image postprocessing are adopted [20, 22, 27, 28]. However, *D*_*fast*_ is known to be highly instable [16, 18, 19] and has rarely been utilized. In this study, we proposed a new approach (i.e., *Y*_*i*_ fitting) for fitting *D*_*fast*_ with 2CM, and this new approach resulted in substantially improved scan–rescan stability as compared with the conventional biex fitting (Table 2). In the current study, for 2CM, *Y*_*i*_ fitting offered liver *D*_*fast*_ CoV of 0.295 for Dataset‐1 and 0.480 for Dataset‐2. In their scan–rescan reproducibility study, Cieszanowski et al. [29] reported liver *D*_*fast*_ CoV of 0.70 ~ 0.99. In the current study, *Y*_*i*_ fitting offered liver *D*_*fast*_ B‐A 95% limits of agreement of −32.15 to 36.84 (10^−3^ mm^2^/s) for Dataset‐1 and −38.60 to 43.60 (10^−3^ mm^2^/s) for Dataset‐2. Chevallier et al. [27] reported liver *D*_*fast*_ B‐A 95% limits of agreement of −77.2 to 79.0 (10^−3^ mm^2^/s) for the repeatability study and −84.0 to 90.3 (10^−3^ mm^2^/s). Note that an absolute comparison to different literature studies would not be possible due to differences in data acquisition parameters. Another important advantage of using *Y*_*i*_ fitting is that only limited nonzero *b*‐values are required for *D*_*fast*_ fitting. We anticipate that the improved stability with *Y*_*i*_ fitting may offer *D*_*fast*_ to be an applicable IVIM parameter for disease clarification.

Though 3CM is known to better describe liver perfusion and diffusion, it has been generally considered that, at the individual study subject or patient level, 3CM is too instable to be applied [3, 6]. Thus, despite the 3CM fitting being published almost 10 years ago, it has been only rarely applied. In the current study, we proposed a new approach to calculate *F*_*fast*_. When *PF*_*0(biex‐IVIM)*_ was calculated initially according to the biex‐IVIM model with fitting started from *b* = 0, this study confirmed that *PF*_*0(biex‐IVIM)*_ is very similar to *PF*_*total(triex‐IVIM)*_. In Figure 7, *F*_*tot*_ in (A1/A2) are *PF*_*0(biex‐IVIM)*_, and *F*_*tot*_ in (B1/B2) are *PF*_*total(triex‐IVIM)*_. As demonstrated by the figure, the means and distributions of the *F*_*tot*_ measurements obtained by both methods are highly similar. The *PF* related to fast perfusion but without very fast perfusion is calculated according to *Y*_*i*_, and the difference between *PF*_*0(biex‐IVIM)*_ and *PF*_*0(Y2 to Y20)*_ is *F*_*vfast*_. Compared with earlier publications such as the results described by Chevallier et al. [6], the scan–rescan reproducibility for perfusion fraction has been much improved in this study. In fact, the repeatability/reproducibility of 3CM perfusion fraction parameters in the current study is similar to the earlier optimized *PF* measure of 2CM. Zheng et al. [22] described the ICC of 2CM *PF*_0_ to be 0.647 and 0.671, respectively, for two datasets. It is well known that in earlier literature that 2CM *PF* measure has higher measurement stability than 3CM metrics, and *PF* measured by 2CM has been widely applied and has been noted as the most sensitive IVIM parameter in many settings [14, 17]. It is possible that, if we can further increase the number of very low and low *b*‐value data (the total number of *b*‐values was 16 in our current study), 3CM may also be clinically applicable eventually.

The primary goal of our study was to improve the measurement stability of *D*_*fast*_ in 2CM and perfusion fraction parameters for 3CM. However, we are not in a better position to offer a physiological explanation of the different measured perfusion fractions obtained from the *Y*_*i*_ fit, as currently there is no reference standard physiologically [4]. In fact, the definitions of 2CM or 3CM (or even 4CM) are more mathematical, based on DWI signal decay pattern. Moreover, MRI scan parameters, such as TE, can also affect the measured values of IVIM parameters [12, 30, 31].

Our results applied signal averaging within ROIs, which increases the SNR and improves fitting stability, as one of our research interests is to use diffusion MRI to assess liver fibrosis. Liver fibrosis is a diffused disease, and the ROI‐based approach is appropriate. We also additionally tested a pixelwise approach. Our analysis shows that the *Y*_*i*_ fitting method produced *D*_*fast*_ mapping with better quality compared with that derived from conventional fitting (Figure S4), while substantially reducing processing time.

This study analyzed both 3.0 T liver diffusion data (Dataset‐1) and 1.5 T liver diffusion data (Dataset‐2). Lower *D*_*fast*_ was measured at 3.0 T by both conventional fitting and *Y*_*i*_ fitting (e.g., *D*_*fast*_ of 48.8 × 10^−3^ mm^2^/s at 3.0 T and *D*_*fast*_ of 55.5 × 10^−3^ mm^2^/s at 1.5 T, *Y*_*i*_ fitting; Table 2), and higher perfusion fraction components were measured at 3.0 T by both conventional fitting and *Y*_*i*_ fitting (e.g., *F*_*tot*_ of 0.286 at 3.0 T and *F*_*tot*_ of 0.220, *Y*_*i*_ fitting; Table 3). These results are consistent with experimental data reported by Riexinger et al. [13] and Cui et al. [32] and are consistent with the meta‐analysis results reported by Li et al. [33]. The explanation of these observations could be complex, but this could be partially due to the fact that the liver at 3.0 T has a shorter T2 relaxation time, which is associated with faster signal loss measured at low *b*‐value [12, 31]. Why *D*_*fast*_ is lower at 3.0 T than at 1.5 T should be explored in future studies.

In recent literature, alternative methods, including Bayesian and deep learning–based approaches, have shown to improve estimation reliability—especially for perfusion‐related parameters [34]. Studies also proposed unsupervised deep neural networks for three‐exponential IVIM modeling in the liver [35, 36]. In our study, we studied the comparisons with the conventional NLLSQ method and the basic tri‐exponential fitting approach, as the DWI images and statistical parameters used in those approaches are closely aligned with ours, allowing for a matched comparison of parameter repeatability. We also attempted to apply the Bayesian fitting method to *Dataset‐1* as a comparative method. However, the quality of Bayesian fitting heavily depends on the performance of the Markov chain Monte Carlo (MCMC) sampling process. We implemented the MCMC chain using a combination of the widely adopted “slicesample” and “ksdensity” functions in MATLAB. Our results indicated that Bayes fitting did not demonstrate superior repeatability relative to the conventional approach. Moreover, achieving higher fitting accuracy for Bayesian fitting requires a substantially larger number of sampling points. Regarding deep learning methods, it requires physiological parameters as a reference to train the model, especially for the supervised methods, and it requires a lot of training data to obtain a robust model. In theory, the *Y*_*i*_ fitting allows for the incorporation of alternative fitting techniques—such as Bayesian methods or neural networks—to estimate the perfusion fraction *PF*_*i*_ values at each sampling point. Improving the accuracy of each *PF*_*i*_ estimation would consequently enhance the quality of the final *Y*_*i*_ fitting result. A key objective of this study is to demonstrate that when fitting the same set of DW images using the IVIM model, the choice of *b*‐values, particularly the starting point of the fitting range, can significantly influence the extracted information and the resulting parameter estimates. The *Y*_*i*_ method was developed to integrate information from different data points with different *b*‐values, thereby reducing sensitivity to the choice of initial fitting conditions and improving the robustness of the derived parameters. Thus, the *Y*_*i*_ fitting approach is not only a specific fitting algorithm but also a novel framework that offers fresh perspectives for measuring IVIM parameters.

There are limitations to the current study. For the 2CM, we applied the conventional fitting to calculate *D*_*slow*_, and with the *Y*_*i*_ fitting approach, we obtained a more reproducible *D*_*fast*_. However, *PF*_*Yi*_, i.e., *PF* calculated with the *Y*_*i*_ approach, did not appear to offer an advantage compared with the conventional fitting (Figure S5). For the 3CM, only *F*_*fast*_ is a unique result calculated with *Y*_*i*_ fitting, whereas *F*_*tot*_ was calculated with conventional fitting, and *F*_*vfast*_ was calculated based on *F*_*tot*_ and *F*_*fast*_. For 3CM testing, an advantage for fast fraction calculation was shown with *Dataset‐2*, but not for *Dataset‐1*. However, our results still suggest that *Y*_*i*_ fitting was not inferior to conventional fitting but may offer better reproducibility in some scenarios, which may depend on IVIM data quality. Moreover, it is interesting to note that, in this study, the ratio of *F*_*fast*_ to *F*_*vfast*_ derived from *Y*_*i*_ fitting is more consistent with the literature results [12] than the ratio of *F*_*fast*_ to *F*_*vfast*_ derived from conventional triex fitting. In this study, the reported *b*‐values were nominal values, as it was difficult to establish the effective *b*‐values in our experiments. Gradient switching for spatial encoding in the slice selection and readout directions always generates *b*‐values greater than 0; this makes it impossible to acquire images with exactly *b* = 0 s/mm^2^. *b*‐values of 0, 2, 4, 7, and so forth should be considered approximations rather than precise values. However, the effect difference between *b* = 0 s/mm^2^ image and *b* = 1 (or 2 s/mm^2^) image has been well noted to be “substantial” [37, 38], suggesting that nominal *b* = 0 s/mm^2^ is indeed much smaller than nominal *b* = 2 s/mm^2^ (or *b* = 1 s/mm^2^). Liver blood vessels including subpixel microvessels show a high signal when there is no motion probing gradient (*b* = 0 s/mm^2^) and a low signal when even very low *b*‐values (such as *b* = 1 and *b* = 2 s/mm^2^) are applied. Overall, liver parenchyma appears to be “brightly gray” on *b* = 0 s/mm^2^ image and “darkly gray” on *b* = 1 or 2 s/mm^2^ images [37, 38]. DW images often suffer from low signal‐to‐noise ratio, and liver DWI particularly suffers from respiratory motion. As shown in Figure 1, in this study, there were steps where volunteers' imaging data with unsatisfactory IVIM fitting or unsatisfactory *Y*_*i*_ fitting were excluded. Poor IVIM fitting excluded 3/39 scans for *Dataset‐1* and 2/42 scans for *Dataset‐2*, and poor *Y*_*i*_ fitting further excluded 4/36 scans for *Dataset‐1* and 3/40 scans for *Dataset‐2*. Although the judgment of “poor fitting” is associated with a certain level of subjectivity, the finally included volunteers' data for repeatability/reproducibility comparison experienced the same level of selection for both fittings. How the *Y*_*i*_ fitting can offer an advantage in classification for various pathological entities will require additional studies. A number of studies have demonstrated the principle that 3CM IVIM may offer advantages over 2CM IVIM [7, 9, 10]. However, the current study only focused on the technical improvement of data fitting stability, whereas the potential benefit for disease classification was not studied.

In summary, this study confirms that biex‐IVIM fitting starting from a lowest *b*‐value of ≤ 10 s/mm^2^ follows a typical exponential pattern for the liver, whereas a *b*‐value of 2 s/mm^2^ or 4 s/mm^2^ is favored for data fitting. The *Y*_*i*_ fitting approach for *D*_*fast*_ in 2CM and perfusion fractions of 3CM can improve data fitting stability and potentially allow better disease classification. The practical application of this *Y*_*i*_ fitting approach with patients' data will be topics of further research.

## Author Contributions

F.‐Z.M. and Y.X.J.W.: conceptualization. F.‐Z.M. and Y.X.J.W.: methodology. Y.X.J.W.: data curation. F.‐Z.M.: formal analysis. Both authors: writing – original draft. Both authors: writing – review and editing. Y.X.J.W.: supervision. Both authors have read and approved the final version of the manuscript.

## Funding

This work was supported by the Hong Kong Government (14112521).

## Conflicts of Interest

The authors declare no conflicts of interest.

## Supporting information


**Figure S1:** Three examples of volunteer scans excluded from final analysis due to unsatisfactory *Y*_*i*_ fitting.
**Figure S2:** Linear regression results of *Y*_*i*_ when fitted with *b* = 2, 4, 7, 10, 15, and 20 s/mm^2^, results for Dataset‐2.
**Figure S3:** Residuals (actual value–predicted value) with the linear fit of *Y*_*i*_ value of *b* = 2, 4, 7, 10, 15, and 20 s/mm^2^, results for Dataset‐2.
**Figure S4:** A comparison of upper abdomen *D*_*fast*_ pixelwise maps constructed by conventional NLLSQ fitting and by *Y*_*i*_ fitting.
**Figure S5:** Perfusion fraction estimation with the two‐compartment model and with *Y*_*i*_ fitting. Testing results for Dataset‐1.

## Data Availability

The data that support the findings of this study are available from the corresponding author upon reasonable request.
