# Supplementary material for: A New Approach for Estimating Liver IVIM Fast Diffusion Coefficient in Two‐Compartment Model and Fast Diffusion Fraction in Three‐Compartment Model: Theory and In Vivo Validation of Two Centers' Data
Source: NMR Biomed. 2025 Dec 29;39(2):e70221. doi: 10.1002/nbm.70221 (PMC12748020; doi:10.1002/nbm.70221)
Supplement: Supplementary file 1 — Figure S1: Three examples of volunteer scans excluded from final analysis due to unsatisfactory Y i fitting. Figure S2: Linear regression results of Y i when fitted with b = 2, 4, 7, 10, 15, and 20 s/mm2, results for Dataset‐2. Figure S3: Residuals (actual value–predicted value) with the linear fit of Y i value of b = 2, 4, 7, 10, 15, and 20 s/mm2, results for Dataset‐2. Figure S4: A comparison of upper abdomen D fast pixelwise maps constructed by conventional NLLSQ fitting and by Y i fitting. Figure S5: Perfusion fraction estimation with the two‐compartment model and with Y i fitting. Testing results for Dataset‐1. [file NBM-39-e70221-s001.docx]

***
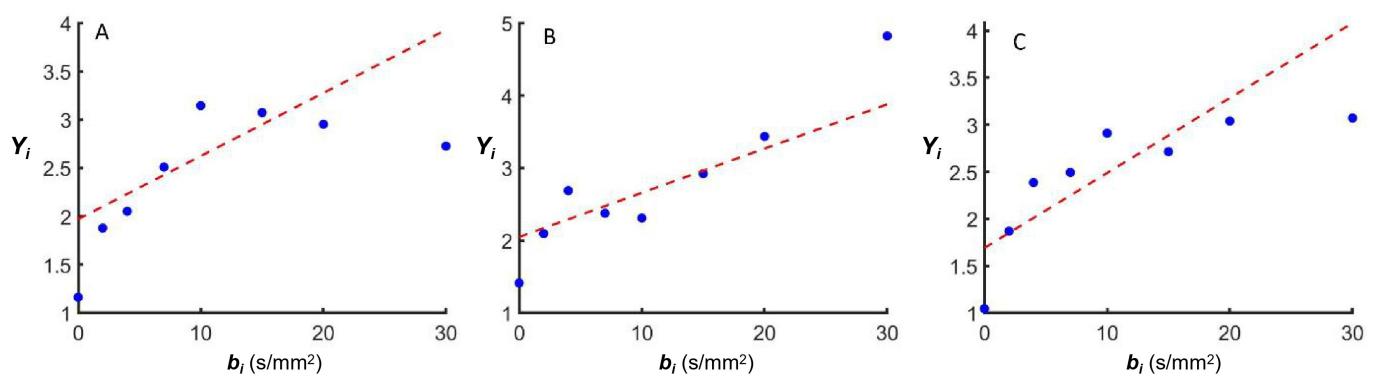
***

***Supplementary Figure 1.*** Three examples of volunteer scans (A, B, C) excluded from final analysis due to unsatisfactory *Y_i_* fitting.


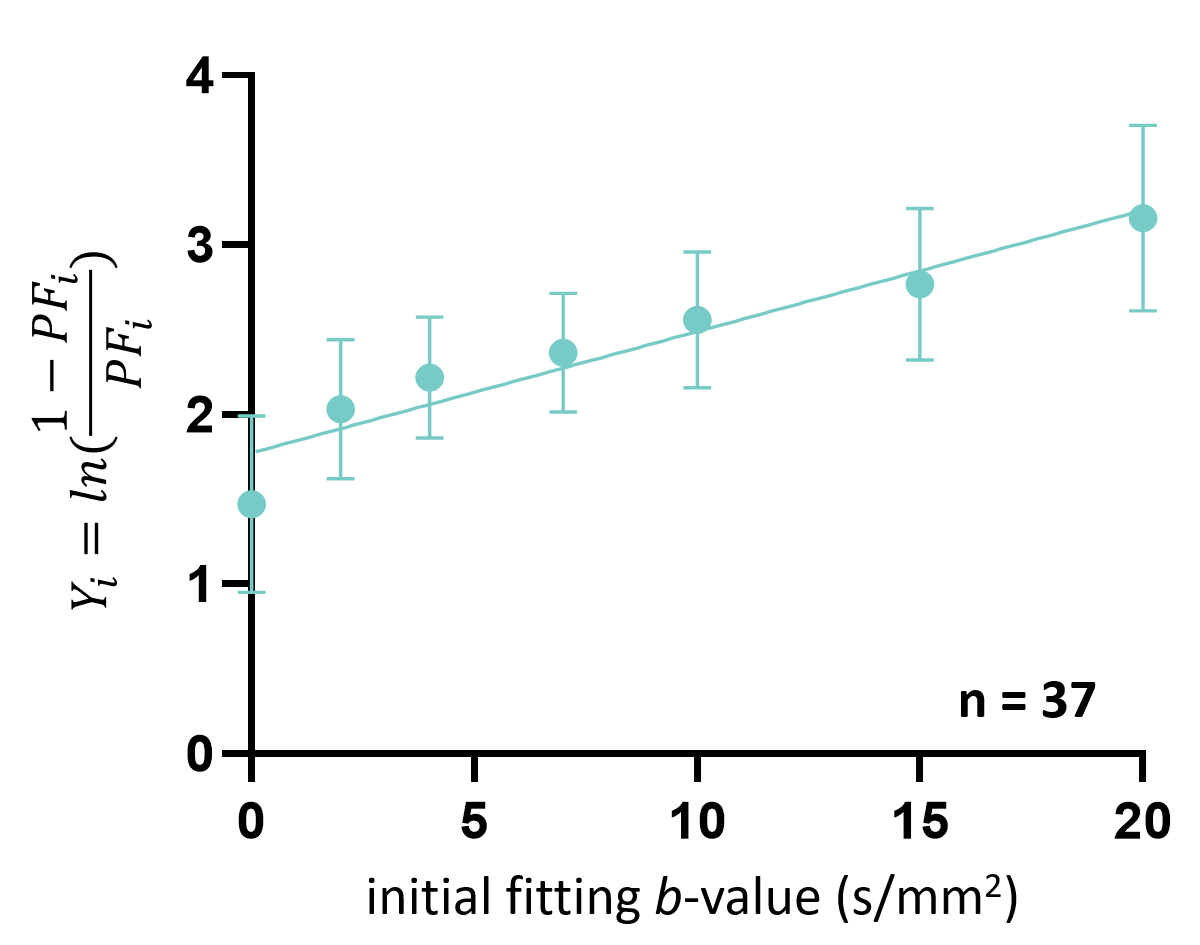


***Supplementary Figure 2*,** Linear regression results of *Y_i_* when fitted with *b*=2, 4, 7, 10, 15, 20 s/mm^2^. Six *Y_i_* values corresponding to *b*= 2, 4, 7, 10, 15, and 20 s/mm^2^ can be linearly fitted, while the *Y_i_* value corresponding to *b*= 0 s/mm^2^ does not correlate with the linear fitting results of other b values. *Y_i_* are presented with mean and standard deviation of the 37 scans in *dataset-2*. n: number of scans.


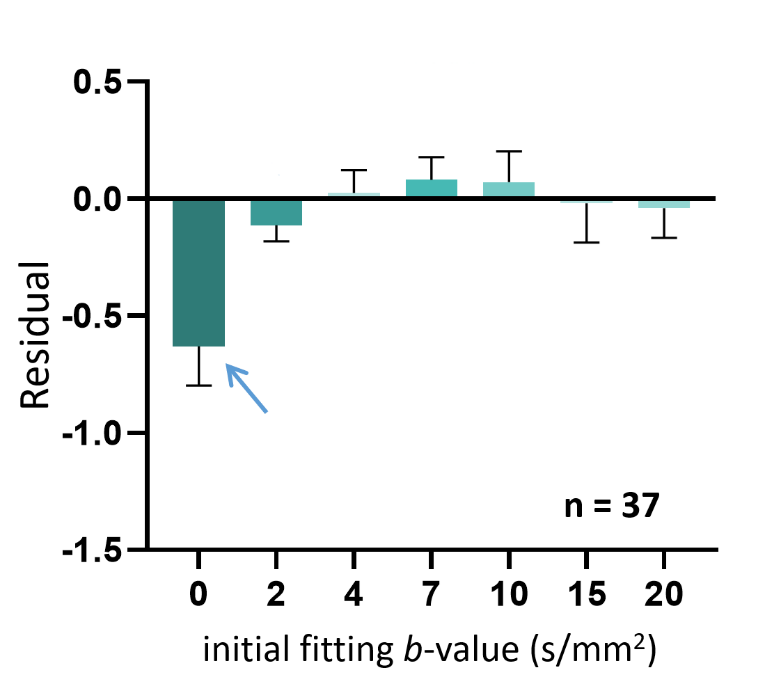


***Supplementary Figure 3*,** Residuals (actual value - predicted value) with the linear fit of *Y_i_* value of *b*= 2, 4, 7, 10, 15, 20 s/mm^2^. The results are based on *dataset-2*, the length of the box at each *b*-value represents the mean of residuals of all 37 linear fittings, and the bar at each box denotes the 95 % CI of the mean value of residual. Residuals at *b*=0 are substantially larger than residuals at other *b*-values. Note that the residual at *b*=2 s/mm^2^ is larger than other higher *b*-values, suggesting that the signal intensity at *b*=2 s/mm^2^ exceeds the value predicted by the bi-exponential model. n: number of scans


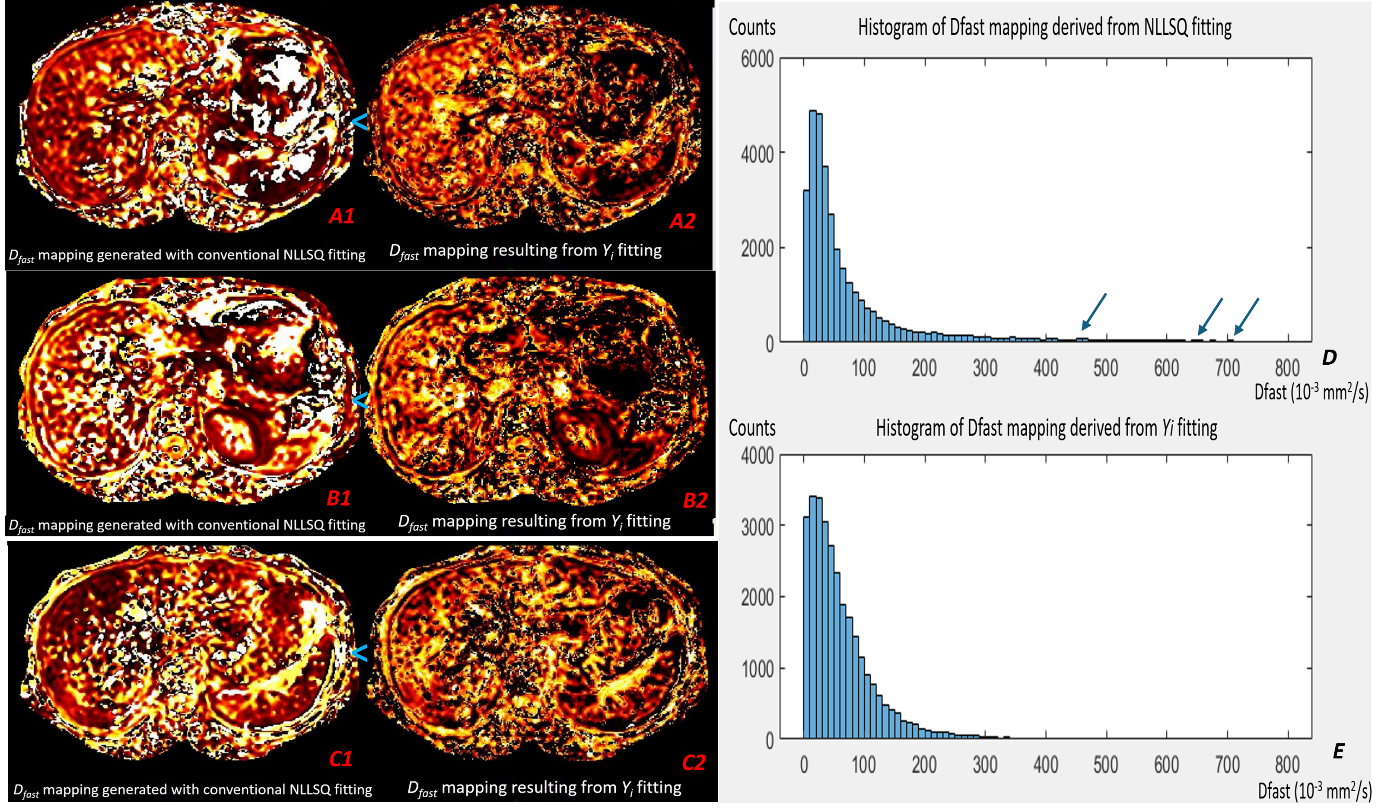


***Supplementary Figure 4.*** Visual comparison of liver pixelwise maps constructed by conventional NLLSQ fitting (A1, B1, C1) and *Y_i_* fitting (A2, B2, C2). There are numerous failed fitting voxels with conventionally fitted map (shown as white pixels), while there are few unaccepted pixels with *Y_i_* fitted map. The distribution of these very high values is unreasonable according to anatomical structure of the upper abdomen, thus the *Y_i_* method offers better fitting quality (denoted by the sign ‘<’). Histogram analysis shows there are extremely high *D_fast_* values calculated with conventional segmented fitting (D, arrows), while there are few such values calculated with *Y_i_* fitting (E).


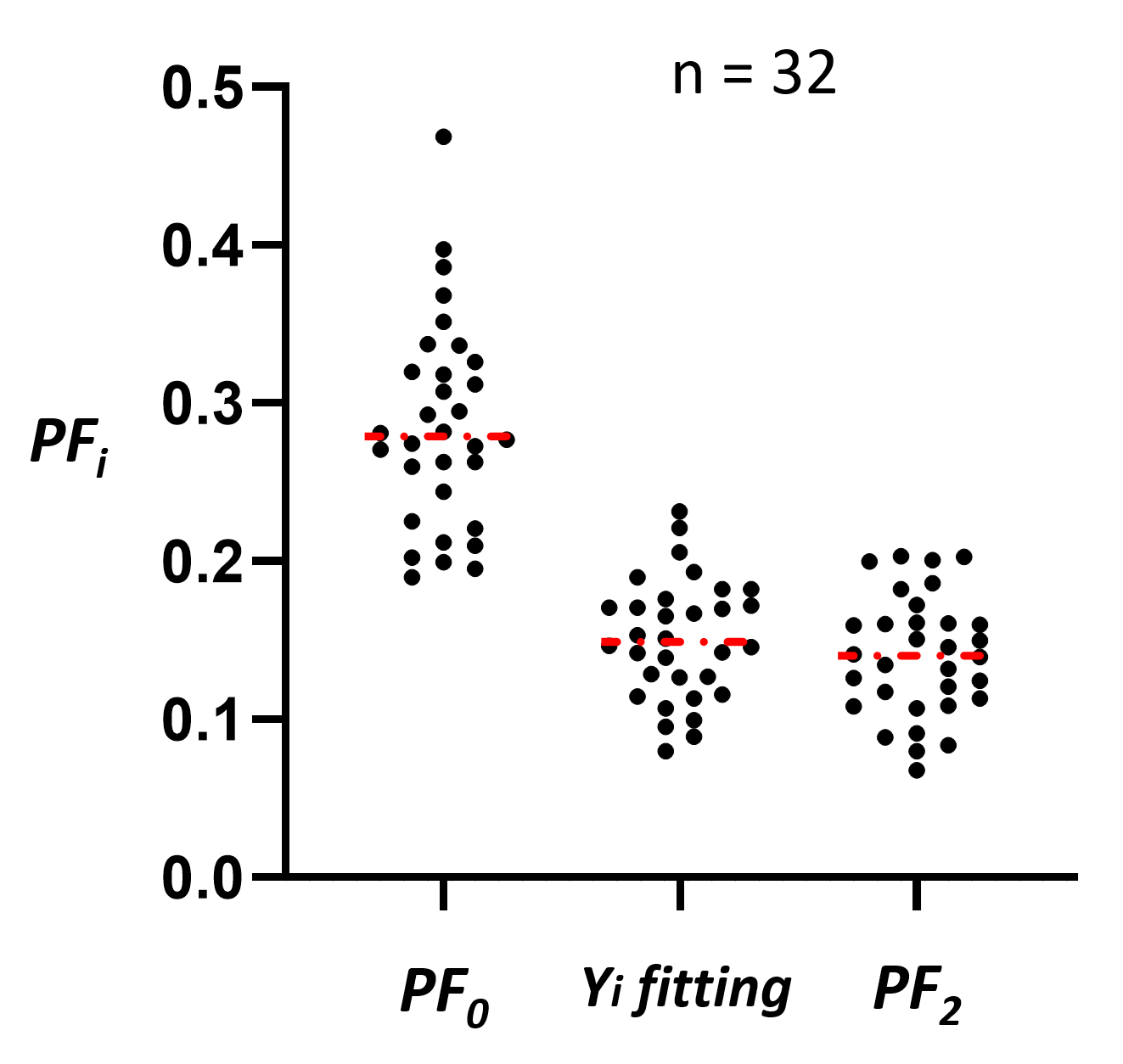


***Supplementary Figure 5*,** Perfusion fraction estimation with the two-compartment model with dataset-1 (n=32). *PF_0_*: *PF* measured with conventional biex-model, and the fit-starting *b*-value was set to 0 s/mm^2^. For *PF_0_*, mean value equals 0.2861, standard deviation (SD) is 0.0653. *PF_2_*: PF measured with standard IVIM model, and the starting fitting b-value was set to 2 s/mm^2^. For *PF_2_*, mean value equals 0.1398, SD is 0.0380. *Y_i_* fitting results was calculated according to Equation (9), and mean value equals 0.1504, SD is 0.0381. *PF_2_* from *Yi* fitting has a CoV of 25.3%; *PF_2_* from conventional biex-IVIM model has a CoV of 27.2%. No apparent advantage was noted for *Yi* fitting. n: number of scans
